# The Zinc Nutritional Immunity of *Epinephelus coioides* Contributes to the Importance of *znuC* During *Pseudomonas plecoglossicida* Infection

**DOI:** 10.3389/fimmu.2021.678699

**Published:** 2021-05-04

**Authors:** Lixing Huang, Yanfei Zuo, Yingxue Qin, Lingmin Zhao, Mao Lin, Qingpi Yan

**Affiliations:** ^1^ Fisheries College, Key Laboratory of Healthy Mariculture for the East China Sea, Ministry of Agriculture, Jimei University, Xiamen, China; ^2^ Fisheries College, Fujian Engineering Research Center of Aquatic Breeding and Healthy Aquaculture, Jimei University, Xiamen, China

**Keywords:** *Pseudomonas plecoglossicida*, *Epinephelus coioides*, dual RNA-seq, *znuC*, nutritional immunity

## Abstract

Previously, the dual RNA-seq was carried out in a *Pseudomonas plecoglossicida*- *Epinephelus coioides* infection model to investigate the dynamics of pathogen-host interplay *in vivo. ZnuC*, a member of ZnuCBA Zn importer, was found transcriptionally up-regulated during infection. Thus, this study aimed to assess its role during the trade-off for Zn between host and *P. plecoglossicida*. ICP-MS analysis and fluorescent staining showed that Zn was withheld from serum and accumulated in the spleen, with increased Zn uptake in the Golgi apparatus of macrophages after infection. Additionally, growth assay, macrophage infection and animal infection after gene knockout / silencing revealed that *znuC* was necessary for growth in Zn-limiting conditions, colonization, intracellular viability, immune escape and virulence of *P. plecoglossicida*. Further analysis with dual RNA-seq revealed associations of host’s Zn nutritional immunity genes with bacterial Zn assimilation genes. *IL6* and *ZIP4* played key roles in this network, and markedly affected *znuB* expression, intracellular viability and immune escape, as revealed by gene silencing. Moreover, EMSA and GFP reporter gene analysis showed that Fur sensed changes in Fe concentration to regulate *znuCBA* in *P. plecoglossicida*. Jointly, these findings suggest a trade-off for Zn between host and *P. plecoglossicida*, while *ZnuC* is important for *P. plecoglossicida* Zn acquisition.

## Introduction

Manganese (Mn), zinc (Zn) and iron (Fe), as well as other transition metals, are vital to living beings, because they play important roles in protein structure and function (1). Pathogenic microorganisms must obtain these micronutrients from the host, while the latter attempts to intercept them through a process called nutritional immunity (2–5).

Fe is a common co-factor, which plays an important role in various physiological processes. Therefore, almost all bacterial pathogens need Fe, whose availability is limited by vertebrates to take advantage of this demand as an effective defense against infection (4, 6). Although the systems by which bacteria obtain Fe and the mechanism of vertebrate host interception of Fe from invading bacteria have been studied longer and more extensively (7–10), a large body of literature exists for other metals.

Zn also plays vital roles in bacteria (5). As the second most abundant transition metal in the majority of organisms, it has both catalytic and structural functions in proteins (11). Indeed, Zn-binding proteins account for about 4 to 8% of all proteins produced by prokaryote organisms (12). In some bacterial pathogens, the lack of a high affinity Zn transport system leads to reduced virulence (4). In the light of these important roles of Zn in bacterial physiology, it is not surprising that Zn sequestration represents an essential innate defense strategy. A typical example of Zn restriction is the staphylococcal abscess, which lacks Zn (13, 14).

Recent studies have elucidated some mechanisms of Zn chelation in the host. The S100 protein family comprises multiple calcium-binding proteins found in vertebrates, some of which are related to resistance to infection. S100A7 (psoriasin), secreted by keratinocytes, exerts antimicrobial effects via Zn chelation (15). S100A12 (calgranulin C) can bind Zn as well (16, 17). Calprotectin, a heterodimer comprising S100A8 and S100A9, accounts for about 40% of neutrophil cytoplasmic proteins. It has strong antimicrobial activity against various bacterial and fungal pathogens (13, 18, 19). Calprotectin achieves its antibacterial activity by chelating Mn and Zn (13). It is involved in host defense against *Salmonella typhimurium*, *Staphylococcus aureus* and fungal pathogens such as *Candida* spp. and *Aspergillus* spp (18–21). Nevertheless, some pathogenic organisms have evolved ingenious mechanisms for combating or twisting these inhibitory features.

To counteract nutritional immunity, pathogens have evolved ingenious strategies for acquiring Zn from the host (22, 23). Although not completely defined, the transport of Zn through the outer membrane in Gram-negative bacteria may be driven by the TonB-ExbBExbD system. ZnuD, a Zn-modulated TonB-dependent receptor, has been characterized in *Neisseria meningitidis* and numerous other pathogens (24). ZnuD is similar to HumA, a *Morexalla catarrhalis* heme transporter. Z*nuD* expression in *Escherichia coli* promotes the acquisition of heme (25). The dual regulation of ZnuD by Zur and Fur (Zn and Fe uptake regulators, respectively) further indicates ZnuD may be involved in the acquisition of Zn and heme (25). In addition, the cross regulation of ZnuD may be due to the increased demand for exogenous heme under Zn limitation, because some endogenous heme biosynthesis enzymes need Zn (26). In Gram-positive and Gram-negative bacteria, Zn import through the plasma membrane is mainly promoted by the ABC-family of transporters, such as ZnuABC (5, 27–30). The importance of Zn transporters to overcoming nutritional immunity and calprotectin has been demonstrated for several pathogens. For example, *Pseudomonas aeruginosa* relys on the znuABC Zn transporter to overcoming calprotectin-mediated growth inhibition (31). Recent studies have shown that the ZnuABC Zn uptake system of *S. typhimurium* is necessary for resistance to Zn chelation mediated by calprotectin accumulation in the intestine after infection (20). In addition, *S. typhimurium* uses calprotectin-mediated Zn-chelation to compete with the host microbiota, which is not well adapted to nutritional deficiency (20).

The mechanism of Zn nutritional immunity has not been fully elucidated. In addition, the majority of existing research evaluating nutritional immunity and the competition for micronutrients between pathogen and host have been carried out in mammalian and human pathogenic bacteria. For example, many *P. aeruginosa* resistance genes were induced during human infection, including those involved in zinc transport (32). At present, studies assessing fish nutritional immunity and its function in pathogen-host interactions are scarce. Through some strategies, it is possible to protect fish from disease by providing balanced food that increase immunity (33). Thus, it is necessary to understand the interaction between the bacterial pathogens and fish host, as well as the most important reasons that can increase or suppress nutritional metal homeostasis.

*Pseudomonas plecoglossicida* is a marine pathogenic bacterium, which can infect *Plecoglossus altivelis (*Ayu) (34), *Pseudosciaena crocea* (large yellow croaker) (35), *Epinephelus coioides* (orange spotted grouper) (36), and *Oncorhynchus mykiss* (rainbow trout) (37). Outbreaks of *P. plecoglossicida* infections in *P. crocea* and *E. coioides* are characterized by internal organ granulomas, and cause severe economic losses. Furthermore, as there is no effective prevention and control measures for this fish infectious disease, there is a risk of antibiotic abuse. “No antibiotics” is the future trend of aquaculture, so it is urgent to find a safe, efficient and healthy method to control *P. plecoglossicida* infections. Our previous dual RNA-seq analysis showed that *znuC* is significantly up-regulated during infection by *Pseudomonas plecoglossicida*, which indicated a critical role for *znuC* during infection (38). In several bacterial pathogens, as a member of the high-affinity Zn transporter ZnuCBA, *znuC* is necessary for virulence and overcoming Zn nutritional immunity. However, its contribution to infection, Zn import and resistance to nutritional immunity have not been experimentally demonstrated. Thus, this study aimed to assess its role during the trade-off established between the host and *P. plecoglossicida* in terms of Zn utilization. The research on the molecular mechanism will contribute to the formulation of the disease control strategy on the basis of nutritional immunity and the development of highly effective live attenuated vaccine.

## Materials and Methods

### Bacteria and Culture Conditions

*P. plecoglossicida* (NZBD9) was obtained from *Pseudosciaene crocea* spleen after natural infection, and confirmed for pathogenicity by artificial infection (35). Storage was performed in saline containing 10% glycerol at −80°C. NZBD9 was cultured in LB (Luria Bertani) medium (18 or 28°C;, 220 rpm). *E. coli* DH5α (TransGen Biotech, China) was also cultured in LB medium (37°C; 220 rpm).

### Generation of *P. plecoglossicida* Mutant Strains and Their Complements

Gene knockout was performed according to previous research (39). The pKD46 plasmid was introduced into *P. plecoglossicida*, and induced by 30 mM L-arabinose (40). The targeted fragments with the termini showing homology to the 50-bp upstream and downstream the flanking regions of *znuC* and *fur*, respectively, were constructed by PCR amplification and introduced into *P. plecoglossicida* via electroporation. Tetracycline (10 μg / mL) was utilized for screening, and clones were verified by PCR. All primers utilized are described in **Table S1**.

To construct the complements for Δ*znuC* and Δ*fur*, the *znuC* and *fur* genes were amplified by PCR and then cloned into the linearized pBAD33 vector using T4 DNA ligase (New England Biolabs) based on the manufacturer’s recommendations. The vectors were transferred into Δ*znuC* and Δ*fur*, and Chloramphenicol was used to screen the positive clones.

### Generation of *P. plecoglossicida* RNAi Strains

RNAi was performed based on a previous report (41). Five short hairpin RNAs against *znuC* and *fur* were provided by Shanghai Generay Biotech (China), respectively. *NsiI* and *BsrGI* (New England Biolabs, USA) were used to linearize the pCM130/tac vector. Then, T4 DNA ligase (New England Biolabs) was used to anneal the oligonucleotides and ligate them to linearized pCM130/tac (42). Through heat shock, recombinant pCM130/tac plasmids were firstly transferred into DH5α. Then, the recombinant pCM130/tac plasmids were purified and introduced into *P. plecoglossicida*. Positive clone screening was performed with tetracycline (10 μg/mL). Finally, quantitative real time PCR (qRT-PCR) was undertaken for validating *znuC* and *fur* expression in RNAi strains.

### Infection and Sample Collection

Healthy *E. coioides* (47.0 ± 2.0 g) were randomized to multiple groups (n=40/group, triplicate assays were performed). Intraperitoneal injection of *P. plecoglossicida* was administered to each fish at 10^3^ CFU/g (43). The negative control group received the injection of sterile PBS. Daily observation was carried out.

For dual RNA-Seq, spleen specimens from six weight-matched *E. coioides* after *P. plecoglossicida* infection were collected at 48 h post-infection (hpi); two consecutive samples were pooled (44). For bacteria distribution assessment, spleens from 3 *E. coioides* were obtained at 1, 6, 12, 24, 48, 72 and 96 hpi, respectively (45, 46). Spleens and blood specimens from 3 *E. coioides* were collected at 48 hpi, for Zn concentration measurements by inductively coupled plasma mass spectrometry (ICP-MS).

Experiments involving animals were performed as recommended by the “Guide for the Care and Use of Laboratory Animals” put forth by the National Institutes of Health. The involved protocols had approval from the Animal Ethics Committee of Jimei University (JMULAC201159).

### Preparation of *E. coioides* Macrophages

*E. coioides* macrophages were isolated as previously described (47). *E. coioides* were anaesthetised with 4-ethyl-amino-benzocaine, and the head-kidneys were sampled, pushed through a 100 mesh nylon screen and then suspended in L-15 medium (Biological Industries, Israel) supplemented with 100 IU streptomycin/penicillin (S/P)/mL, 10 IU/mL heparin and 2% foetal calf serum (FCS). The cell suspension was layered onto a 34%/51% discontinuous Percoll (Amersham Pharmacia Biotech, UK) density gradient with a syringe and centrifuged at 400×g for 30 min at 4 °C. The band of cells in the layer above the 34%/51% interface was collected, washed twice and re-suspended with L-15 medium with 100 IU S/P/mL, 10 IU heparin/mL and 10% FCS. The cells were then incubated at 28 °C. After 4 h, non-adherent cells were removed by washing with L-15 and monolayers were collected. Then, the cell suspension was adjusted to ~2×10^6^ cells/mL in L-15 medium with 100 IU S/P/mL, 10 IU heparin/mL and 10% FCS and transferred to 6-well plates at 1 mL/well.

### Macrophage Nucleofection With siRNA

Macrophage nucleofection with siRNA was carried out based on a previous report (48). Small interfering RNAs (siRNAs) were manufactured based on target gene sequences by GenePharma (China) (**Table S1**). Macrophages (10^7^/mL) were cultured in L-15 medium (PAN-Biotech GmbH, Germany) with 10% fetal calf serum. 2 µl of each siRNA and 20 µl per 200,000 cells suspended in Nucleofector™ solution SF were then added to each well of a sterile 96-well plate to be transfected. Then the nucleofection was carried out on an Amaxa nucleofector 96-well shuttle system according to the manufacturer’s recommendations. qRT-PCR and *P. plecoglossicida* infection were performed at 24 h post-nucleofection.

### Cell Infection With *P. plecoglossicida*

Macrophages from *E. coioides* head-kidney were isolated as the method described by Zhang et al. (47) and seeded in a 6-well culture plate which contained 2 mL of L-15 medium supplemented with 10% FCS and 100 IU/mL of S/P in each well. After incubation at 28°C for 4h, the cultures were washed twice and the macrophages (10^7^ cells/mL) were incubated in L-15 medium containing 10% FCS with the wild-type and mutant/knock-down strains of *P. plecoglossicida* [multiplicity of infection (MOI)]=100 (100 bacteria per macrophages added). After incubation at 18°C for 1 h, the macrophages were washed twice with cold PBS, re-suspended in 2 mL PBS, treated with 250 mg/mL gentamycin for 20 min at 18°C to eliminate extracellular bacteria, and then washed twice with PBS. The supernatant was withdrawn and tested for sterility. The cells were re-suspended in fresh L-15 medium with 10 IU/mL heparin, 100 IU/mL S/P, and 10% FCS, and this time point was denoted as 0 h. Then, the 0 h samples were further incubated for 1 h and 3 h and the samples were denoted as 1 h and 3 h, respectively. After that, the cells from 0 h to 3 h samples were centrifuged for 5 min at 100×g at 18 °C, and 1 mL sterile distilled water was added for 30 min to lyse the cells. The CFU of the precipitate was determined by plating appropriate dilutions on TSA plates. For the 3 h sample, the precipitate of macrophages was removed by centrifugation and the CFU of bacteria in the supernatant was counted on TSA plates. The intracellular survival rate was defined as the number of CFU at 1 h divided by the number of CFU at 0 h. The escape rate was defined as the number of CFU of the supernatant at 3 h divided by the number of CFU at 0 h.

### ICP-MS

20 mg spleen samples were freeze-dried for 24 h at -80°C and homogenized individually with an agate mortar prior to analysis. Then, acid digestion with HNO_3_+H_2_O_2_ (5:1) by microwave-assisted digestion was carried out (49). 500 μL of serum samples were mixed with 500 μL of H_2_O_2_ and 1 mL of 14 M HNO_3_ in PET bottles and diluted to 30 g with MilliQ deionized water. Zinc content was determined by ICP-MS (Agilent 7700x) and normalized to mass and volume, respectively.

### Fluorescent Staining

*E. coioides* macrophages were infected with *P. plecoglossicida* for 24 h followed by incubation with Golgi and Zn dyes for 30 min (50). Zinquin ethyl ester (MKBio, China) was used for the visualization of labile Zn. BODIPY TR Ceramide (MKBio, China) was used for the staining of Golgi. Images were generated with a LEICA SP8 confocal microscopy.

### DNA Isolation

The Wizard genomic DNA purification kit (Promega, USA) was utilized for genomic DNA extraction (51).

### RNA Isolation

Total RNA extraction used TRIzol reagent (Invitrogen, USA) as we described before (52, 53). The mixed genomic DNA in total RNA was digested with the Turbo DNA-free DNase (Ambion, Austin, TX, USA). The RNA quality was assessed using an Agilent 2100 Bioanalyzer (Agilent Technologies, Santa Clara, CA, USA). The Ribo-Zero rRNA removal kit (Epicentre, USA) was utilized for rRNA removal.

### qRT-PCR

Gene expression was detected on a QuantStudio 6 Flex real-time PCR system (Life Technologies, USA), with *gyrB* and *β-actin* used as reference genes for *P. plecoglossicida* and *E. coioides*, respectively (54–56). The 2^−ΔΔCt^ method was applied for analysis. The number of *gyrB* DNA copies/milligram of tissue was utilized for dynamically estimating *P. plecoglossicida* distribution in host spleen (57). **Table S1** lists all primers.

### Transcriptomic Analysis

#### Library Preparation and Sequencing

RNA-seq library preparation utilized the TruSeqTM RNA sample preparation kit (Illumina, USA). Then, rRNA-free RNA samples were incubated with fragmentation buffer, and the SuperScript double-stranded cDNA synthesis kit (Invitrogen) was employed for cDNA synthesis. Amplification utilized Phusion DNA polymerase (New England Biolabs), and Illumina HiSeq4000 sequencing was then performed by Majorbio Biotech (China) (58). The PE150 sequencing strategy was selected, with a 250-300 bp insert strand specific library.

#### Read Processing and Mapping

Sickle (Version 1.33) and SeqPrep (Version 1.3.2-2) were used for the trimming and quality control of raw reads. With Bowtie2 (2.4.2), clean reads mapping to the genome of *P. plecoglossicida* strain NZBD9 [NCBI Sequence Read Archive (SRA); accession number SRP062985] was carried out (59). Mapped reads were considered to belong to *P. plecoglossicida*, and the remaining ones underwent *de novo* assembly to yield *E. coioides* unigenes (58). The summary of the number of mapped reads/input reads per replicate and the coverage have been listed in **Table S2**.

#### *De Novo* Assembly and Host mRNAs Annotation

Clean reads not mapped to *P. plecoglossicida* genome from spleen specimens after infection with wild-type and RNAi strain, respectively, were considered a pool of reads. Then, *de novo* assembly into unigenes was carried out with Trinity (Version 2.12.0) using a kmer size of 25 (60). The bacterial NCBI non-redundant (NR) protein database was utilized for removing any possible prokaryote contaminants. Clean unigenes were next assessed in various databases such as NCBI NR protein, STRING, SWISS-PROT and Kyoto Encyclopedia of Genes and Genomes (KEGG) with BLASTX for identifying proteins showing highest sequence similarities with the obtained unigenes. Blast2GO was utilized for Gene Ontology (GO) analysis (61), and KEGG was employed for metabolic pathway analysis (62).

#### Differentially Expressed Gene (DEG) Determination

Based on NCBI (NZ_ASJX00000000.1) annotations and the abovementioned reference transcriptome annotation, analysis of *E. coioides* transcriptome was carried out. DEGs with | log2FC | ≥ 1 and a false discovery rate (FDR) below 0.05 were tested by the edgeR package (63).

#### Prediction of a Gene Regulatory Network in Pathogen-Host Interactions

With the NetGenerator algorithm (64, 65), a gene regulatory network among *E. coioides* Zn nutritional immunity related DEGs and *P. plecoglossicida* Zn acquisition related DEGs was predicted. Its robustness was assessed, and edges detected by >50% of all iterations were included in the final network.

### Growth Curves

Wild type, *znuC*-50%RNAi, *znuC*-95%RNAi, Δ*znuC* and *znuC* ^+^ strains of *P. plecoglossicida* were cultured in LB medium supplemented with 2 μM Tetrakis-(2-pyridylmethyl)-ethylenediamine (TPEN) overnight (28°C; 220 rpm). The overnight culture was diluted to OD_600_=0.2, and incubated at 18°C; for 24 h while measuring OD_600_ at 0, 3, 6, 9, 12, 15, 18, 21, 24 h (66).

### GFP Reporter Gene System

GFP reporter gene systems were constructed based on previously reported descriptions (67). Briefly, the pET16b-EGFP plasmid containing the Fur binding site was transformed into wild type strain and *fur* knockout/knockdown strains, respectively, via electroporation. The primers used here are listed in **Table S1**.

### Production of Full-Length Fur

Recombinant Fur protein was expressed and purified according to previous descriptions (68). **Table S1** lists all the primers utilized to amplify the full-length of *fur*, which was then cloned into the pET-32a (+) expression vector linearized with *EcoR I* and *Xho I* digestion. The plasmid was then transformed into *E. coli* BL21, and the recombinant Fur protein was induced with isopropyl-β-D-thiogalactopyranoside (IPTG) and purified with a nickel-nitrilotriacetic acid (Ni-NTA) column (TaKaRa).

### Electrophoretic Mobility Shift Assay (EMSA)

EMSA was performed with mixtures of the Fur protein at different levels (0, 0.5, 1, 1.5, and 2 μM) and *znuC* promoter DNA fragments (2 μg) conjugated to 6-carboxyfluorescein at the 5’ terminus (GenePharma) according to previous descriptions (69). The reaction mixture (50 μL) contained 200 mM KCl, 50 mM Tris-HCl, 5% v/v glycerol and 0.1 mM EDTA. The mixture was incubated at 25 °C for 2 h and then loaded (15 μL) onto each lane of a 5% native polyacrylamide gel for electrophoresis (Chakraborty et al., 2010).

### Reverse Transcription PCR (RT-PCR) Studies

Based on the genomic DNA sequence of *P. plecoglossicida*, four pairs of primers were used to determine whether *znuCBA* genes were transcribed into a single operon as previously reported (69–71). **Table S1** lists all the primers utilized.

### Statistical Analyses

Data are mean ± SD, and were compared by one-way ANOVA with post-hoc Dunnett’s test. SPSS 13.0 was used for data analysis. *P*<0.05 indicated statistical significance.

### Data Access

Read data can be retrieved from the SRA database (accession number: PRJNA613574).

## Results

### Effect of *P. plecoglossicida* Infection on Zn Distribution in *E. coioides*

**Figure 1:**
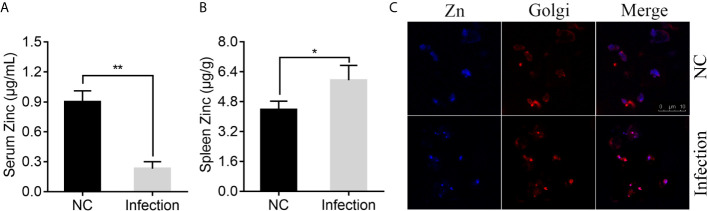
Effect of *Pseudomonas plecoglossicida* infection on Zinc (Zn) concentration in serum, spleen, and macrophages. **(A)** Serum Zn concentrations and **(B)** spleen Zn concentrations were measured via inductively coupled plasma mass spectrometry (ICP-MS) in *Epinephelus coioides* infected with *P. plecoglossicida* at 48 hpi and compared to the healthy *E. coioides*. Values are mean ± SD (n = 3, *P < 0.05, **P < 0.01). **(C)** Confocal microscopy was used to visualize labile Zn with the fluorescent probe, Zinquin ethyl ester. Staining for Zn (blue) and Golgi (red) in macrophages. Purple shows a co-localization of Golgi and Zn. Scale bars represent 10 μm, 3 independent experiments.

To determine whether infection of *P. plecoglossicida* affects the Zn distribution in *E. coioides*, serum and spleen Zn concentrations were measured via ICP-MS in *E. coioides* infected with *P. plecoglossicida* at 48 h post-infection (hpi) and compared to the healthy *E. coioides*. As shown in **Figure 1A**, *P. plecoglossicida* infection produced hypozincemia in *E. coioides*. The serum Zn concentration in *P. plecoglossicida* infected *E. coioides* decreased ~4-fold compared to the healthy *E. coioides*, indicating that Zn was withheld from serum upon *P. plecoglossicida* infection. In the *E. coioides* spleen, a significant increase of Zn was observed after *P. plecoglossicida* infection (**Figure 1B**). The spleen Zn concentration in *P. plecoglossicida* infected *E. coioides* increased by ~1.57 μg/g compared to the healthy *E. coioides*, indicating that Zn was accumulated in spleen upon *P. plecoglossicida* infection.

According to previous study, *P. plecoglossicida* are phagocytosed by macrophages in the spleen of the infected fish, while it is proved to be capable of intracellular survival and replication (72, 73). Macrophages possess numerous mechanisms to combat microbial invasion, including sequestration of essential nutrients, like Zn (50). Therefore, we speculated that Zn nutritional immunity in macrophages may affect the intracellular survival of *P. plecoglossicida*, and detected the distribution of Zn after *P. plecoglossicida* infecting macrophages. Confocal microscopy was used to visualize labile Zn with the fluorescent probe, Zinquin ethyl ester. Uninfected resting macrophages showed labile Zn diffuse distribution in the cytoplasm (**Figure 1C**); however, *P. plecoglossicida* infection caused focal Zn accumulation into the Golgi apparatus (**Figure 1C**), suggesting that *P. plecoglossicida* infection induced Zn redistribution in macrophages, which may limit its accessibility to *P. plecoglossicida*.

Collectively, these data indicated the existence of Zn nutritional immunity induced by *P. plecoglossicida* infection.

### Effect of *znuC* on the Growth in Zn-Limiting Conditions

**Figure 2:**
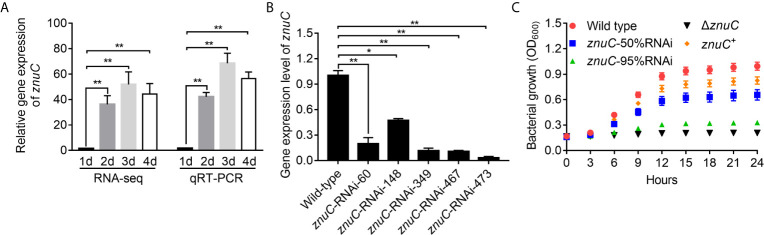
ZnuC is essential for *Pseudomonas plecoglossicida* growth under Zinc (Zn) limiting conditions. **(A)** Expression of *znuC* during infection was determined by dual RNA-seq and quantitative real time PCR (qRT-PCR). **(B)** To characterize *znuC*’s function, five *znuC*-RNAi strains were constructed, and the silencing efficiencies were validated by qRT-PCR. **(C)** To validate whether the *znuC* in *P. plecoglossicida* is important for growth during Zn limitation, growth of wild type, *znuC*-50%RNAi, *znuC*-95%RNAi, Δ*znuC* and *znuC* ^+^ strains were compared in Zn-limiting condition with 2 μM TPEN. *gyrB* was used as a reference gene in the qRT-PCR analysis. Values are mean ± SD (n = 3, *P < 0.05, **P < 0.01).

By dual RNA-seq performed previously (38) and qRT-PCR performed here, we assessed *znuC* expression at 1~4 days after infection. *znuC* was significantly induced from 2 to 4 days after infection, while the peak appeared on the third day, indicating the potential role of *znuC* during *P. plecoglossicida* infection (**Figure 2A**).

To characterize *znuC*’s function, five *znuC*-RNAi strains were constructed, and the silencing efficiencies were validated by qRT-PCR. Among them, *znuC*-RNAi-148 and *znuC*-RNAi-473 strains, with about 50% and 95% *znuC* silencing efficiencies, respectively, were assessed in further studies (**Figure 2B**). Meanwhile, a *znuC* null mutant strain Δ*znuC*, as well as a complemented strain *znuC* ^+^ were constructed (**Figure S1**) and also used to characterize the function of *znuC*.

To validate whether the *znuC* in *P. plecoglossicida* is important for growth during Zn limitation, growth of wild type, *znuC*-50%RNAi, *znuC*-95%RNAi, Δ*znuC* and *znuC* ^+^ strains were compared in Zn-limiting condition with 2 μM Tetrakis-(2-pyridylmethyl)-ethylenediamine (TPEN). The Δ*znuC* mutant grew to a significantly lower terminal optical density than wild type (**Figure 2C**), while the growth defect could be complemented by ectopic expression of *znuC* (**Figure 2C**). After *znuC* knockdown, *P. plecoglossicida* showed significantly decreased growth under Zn limiting culture conditions in a *znuC* dependent manner (**Figure 2C**). These results supported a role for *znuC* of *P. plecoglossicida* in resisting Zn starvation.

### Effect of *znuC* on the Virulence of *P. plecoglossicida*

**Figure 3:**
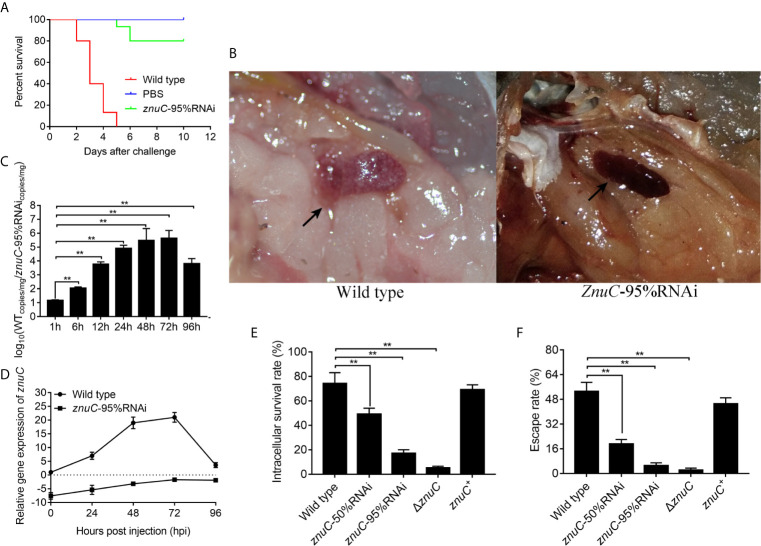
ZnuC is essential for *Pseudomonas plecoglossicida* virulence. **(A)** To assess the importance of *znuC* to *P. plecoglossicida* pathogenesis, *Epinephelus coioides* were infected with *P. plecoglossicida* wild type and *znuC*-95%RNAi. Amounts of *E. coioides* that survived after infection with the indicated strains were compared (n=3). **(B)** The *E. coioides* infected with *P. plecoglossicida* were dissected and observed at 96 hpi. Symptoms of *E. coioides* spleen after infection with *P. plecoglossicida* were compared. **(C)** The bacterial burdens of *P. plecoglossicida* wild type and *znuC*-95%RNAi in *E. coioides* spleen were measured by quantitative real time PCR (qRT-PCR) at 1, 6, 12, 24, 48, 72, and 96 hpi. Temporal dynamic distribution of *P. plecoglossicida* in *E. coioides* spleen were compared. **(D)** To determine whether the reduced bacterial burdens of the *znuC*-95%RNAi in *E. coioides* spleen was due to the stable low expression of *znuC*, we compared the expression of *znuC* in *P. plecoglossicida* wild type and *znuC*-95%RNAi during the infection at 0, 24, 48, 72 and 96 hpi. **(E, F)** Given our observation of increased Zinc (Zn) uptake in the Golgi apparatus of *E. coioides* macrophages after infection, we sought to validate whether the *znuC* in *P. plecoglossicida* is important for intracellular survival. The intracellular viability and immune escape of wild type, *znuC*-50%RNAi, *znuC*-95%RNAi, Δ*znuC* and *znuC* ^+^ strains were compared in *E. coioides* macrophages. Values are mean ± SD (n = 3, **P < 0.01).

To assess the importance of *znuC* to *P. plecoglossicida* pathogenesis, *E. coioides* were infected with *P. plecoglossicida* wild type and *znuC*-95%RNAi. Infection with *znuC*-95%RNAi resulted in significantly delayed death and lower mortality compared to infection with wild type (**Figure 3A**). The initial death time was delayed by 3 days, and the cumulative mortality rate was reduced by 80%. The *E. coioides* infected with *P. plecoglossicida* were dissected and observed at 96 hpi. It was found showed that the spleen specimens from the wild-type *P. plecoglossicida* group had characteristic symptoms (spleen blanketed by a large number of white spots), which were almost inexistent in *E. coioides* administered the *znuC*-95% RNAi strain (**Figure 3B**). These findings indicated that *znuC* was important for *P. plecoglossicida* virulence.

The bacterial burdens of *P. plecoglossicida* wild type and *znuC*-95%RNAi in *E. coioides* spleen were measured by qRT-PCR at 1, 6, 12, 24, 48, 72, and 96 hpi. Spleen amounts of the *znuC*-95% RNAi strain were markedly reduced than those of wild-type *P. plecoglossicida* at most times following infection (**Figure 3C**). To determine whether the reduced bacterial burdens of the *znuC*-95%RNAi in *E. coioides* spleen was due to the stable low expression of *znuC*, we compared the expression of *znuC* in *P. plecoglossicida* wild type and *znuC*-95%RNAi during the infection at 0, 24, 48, 72 and 96 hpi. Within 96 h after infection, *znuC* levels in the spleen were starkly reduced in the *znuC*-95% RNAi strain group in comparison with the wild-type group (**Figure 3D**). These findings indicated that *znuC* was important for *P. plecoglossicida* colonization.

Given our observation of increased Zn uptake in the Golgi apparatus of *E. coioides* macrophages after infection, we sought to validate whether the *znuC* in *P. plecoglossicida* is important for intracellular survival. The intracellular viability and immune escape of wild type, *znuC*-50%RNAi, *znuC*-95%RNAi, Δ*znuC* and *znuC* ^+^ strains were compared in *E. coioides* macrophages. The Δ*znuC* mutant displayed a significantly lower intracellular viability and immune escape than wild type, while the defects could be complemented by ectopic expression of *znuC* (**Figures 3E, F**). After *znuC* knockdown, *P. plecoglossicida* showed significantly decreased intracellular viability and immune escape in a *znuC* dependent manner (**Figures 3E, F**). These results supported a role for *znuC* of *P. plecoglossicida* intracellular survival.

Taken together, these findings illuminated an important role for *znuC* in *P. plecoglossicida* pathogenesis.

### A Dual RNA-Seq-Based Screen Identifies Key Genes Involved in the Zn Trade-Off

In order to simultaneously detect the gene expression patterns of pathogen and host *in vivo*, so as to understand the trade-off established between the host and the pathogen in terms of Zn utilization, especially the role of *znuC* in this process, dual RNA-seq was carried out in *E. coioides* injected with PBS, wild-type or *znuC*-95% RNAi *P. plecoglossicida*.

**Figure 4:**
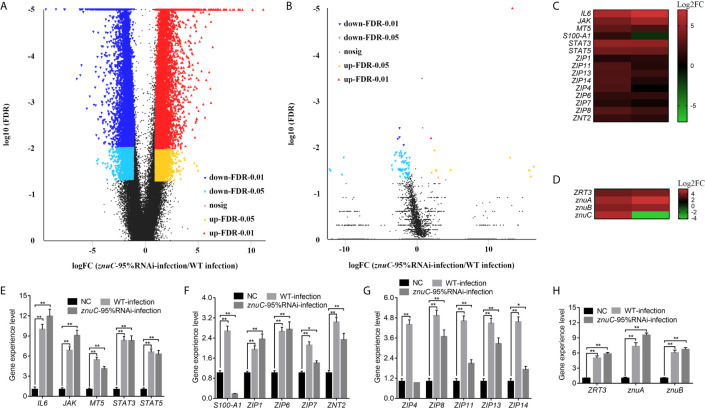
Dual RNA-seq reveals differentially expressed genes (DEGs) associated with the trade-off for Zinc (Zn) between host and pathogen during *Pseudomonas plecoglossicida* infection. **(A, B)** Dual RNA-seq was carried out to assess spleen specimens from *Epinephelus coioides* upon infection with *P. plecoglossicida* for 48h. Compared to the wild-type *P. plecoglossicida* group, spleen specimens from animals infected with the *znuC-*95%RNAi strain had 23,059 DEGs, including 12,005 downregulated and 11,054 upregulated genes. Totally 5,103 genes were detected in the transcriptome of *P. plecoglossicida* from *E. coioides* spleen. In comparison to wild-type *P. plecoglossicida* in *E. coioides* spleen, the *znuC-*95%RNAi strain had 88 DEGs, including 74 downregulated and 14 upregulated genes. Volcano plot indicated upregulated and downregulated genes of *E. coioides* **(A)** and *P. plecoglossicida* **(B)** detected by dual RNA-seq here. **(C, D)** Among all the DEGs, 15 Zn nutritional immunity related genes were identified in *E. coioides*, while 4 DEGs related to Zn acquisition were found in *P. plecoglossicida*. Heat maps indicated *E. coioides* Zn nutritional immunity related genes and *P. plecoglossicida* Zn acquisition genes (adjusted FDR < 0.05; | log2FC | ≥ 1; n=3) compared to healthy *E. coioides*. Upregulated and downregulated genes are colored in red and green, respectively. **(E, F)** quantitative real time PCR (qRT-PCR) analysis of *E. coioides* Zn nutritional immunity related genes and *P. plecoglossicida* Zn acquisition genes in *E. coioides* injected with PBS, wild type and *znuC*-RNAi *P. plecoglossicida*, respectively. Values are mean ± SD (n = 3, *P < 0.05, **P < 0.01).

Dual RNA-seq was carried out to assess spleen specimens from *E. coioides* upon infection with *P. plecoglossicida* for 48h. *E. coioides* transcriptomes after injection of PBS, and wild-type and *znuC*-95% RNAi *P. plecoglossicida* were significantly different. Totally 166517 genes were detected in *E. coioides* spleen. Compared to the wild-type *P. plecoglossicida* group, spleen specimens from animals infected with the *znuC-*95%RNAi strain had 23,059 differentially expressed genes (DEGs), including 12,005 downregulated and 11,054 upregulated genes (**Figure 4A**). These large amounts of differentially expressed genes revealed the important role of *znuC* in *P. plecoglossicida* pathogenicity. According to the latest version of the KEGG database, these 23,059 DEGs were clustered in 56 KEGG pathways, including those tightly associated with immunomodulation, e.g., phagosome, cytokine-cytokine receptor interaction, ECM-receptor interaction, antigen processing and presentation, complement and coagulation cascades, intestinal immune network for IgA production, and platelet activation (**Figure S2**). 108 DEGs, such as *stx13*, *tfr*, *tap*, *sec61*, and *p40phox* were enriched in the phagosome pathway, while phagocytosis is a central mechanism in the inflammation and defense against infectious agents. 91 DEGs, such as *IL8*, *IL8RB*, *CCL4*, *tbfB2*, and *tgfBR2* were enriched in the cytokine-cytokine receptor interaction pathway, while cytokines are crucial intercellular regulators in innate and adaptive inflammatory host defenses, cell differentiation, and repair processes aimed at the restoration of homeostasis. 68 DEGs, such as *reelin*, *thbs*, *tenascin*, *rhamiv*, and *syndecan* were enriched in the ECM-receptor interaction pathway, while these interactions lead to a direct or indirect control of cellular activities such as adhesion, migration, differentiation, proliferation, and apoptosis. 48 DEGs, such as *tnfA*, *hsp70*, *tapbp*, *aep*, and *slip* were enriched in the antigen processing and presentation pathway, whose importance in resisting bacterial infection is self-evident. These findings indicated quite distinct physiological states of the host under infection of *P. plecoglossicida* wild type and *znuC*-95%RNAi. It is worth noting that many immune related genes are up-regulated instead of down regulated in the infection with *znuC*-95%RNAi strain compared to the infection with wild type strain. Thus, we believe that this is not only due to the differential bacterial burdens, which results in less stimulation to the immune system, but also due to the decreased zinc uptake of *znuC*-RNAi strain, which changes the host’s resistance strategies.

Compared to wild-type *P. plecoglossicida*, a large number of genes in *znuC*-95% RNAi strain changed significantly *in vivo*. This may be due to the loss of *znuC* increases the extent of Zn limitation experienced by the bacterium, which suggested the important role of *znuC* during the pathogenesis. Totally 5,103 genes were detected in the transcriptome of *P. plecoglossicida* from *E. coioides* spleen. In comparison to wild-type *P. plecoglossicida* in *E. coioides* spleen, the *znuC-*95%RNAi strain had 88 DEGs, including 74 downregulated and 14 upregulated genes (**Figure 4B**). These 88 DEGs were clustered in 25 KEGG pathways, including those tightly associated with bacterial virulence regulation, e.g., flagellar assembly, bacterial chemotaxis, ABC transporters, bacterial secretion system, two component system and protein export (**Figure S3**). 8 DEGs, including *flgF*, *flgI*, *motB*, *motA*, *fliE*, *FliF*, *FliG*, and *FliS* were enriched in the flagellar assembly pathway, while the flagellar assembly plays important roles in host-microbial interactions, bacterial colonization and virulence. It is worth noting that all these 8 genes were significantly down-regulated in the *znuC*-95%RNAi strain compared to the wild type strain. 4 DEGs, including *fliG*, *cheB*, *motB*, and *motA* were enriched in the bacterial chemotaxis pathway, while bacterial chemotaxis plays a critical role for fitness and virulence during infections. 6 DEGs, including *cysP*, *porH*, *livM*, *metQ* and *metN* were enriched in the ABC transporters pathway, which are responsible for the transport of sulfate, putrescine, branched-chain amino acid, and D-methionine, respectively. It is worth noting that all these 6 genes were significantly down-regulated in the *znuC*-95%RNAi strain compared to the wild type strain, which indicated that there might be a cross-talk between these nutrition transport and the Zn transport. These findings suggested a critical role of *znuC* in the pathogenesis.

Among all the DEGs, 15 Zn nutritional immunity related genes were identified in *E. coioides*, including *IL6*, *JAK*, *MT5*, *S100-A1*, *STAT3*, *STAT5*, *ZIP1*, *ZIP4*, *ZIP6*, *ZIP7*, *ZIP8*, *ZIP11*, *ZIP13*, *ZIP14*, and *ZNT2*. Compared with healthy *E. coioides*, these genes were significantly upregulated in wild-type and *znuC-*95%RNAi strain infected *E. coioides*, except for *S100-A1* that was downregulated in *E. coioides* upon infection with the *znuC-*95%RNAi strain (**Figure 4C**). Meanwhile, only 4 DEGs related to Zn acquisition were found in *P. plecoglossicida*, including *ZRT3*, *znuA*, *znuB* and *znuC* (**Figure 4D**). The results of transcriptome analysis were confirmed by qRT-PCR, further supporting dual RNA-seq’s reliability (**Figures 4E–H**). Since the purpose of this research was to better understand the contention between host and *P. plecoglossicida* for Zn, we selected the above 19 genes to further analyze host-pathogen interactions.

### Prediction of a *znuC*-Dependent Regulatory Network in Pathogen-Host Interactions

**Figure 5:**
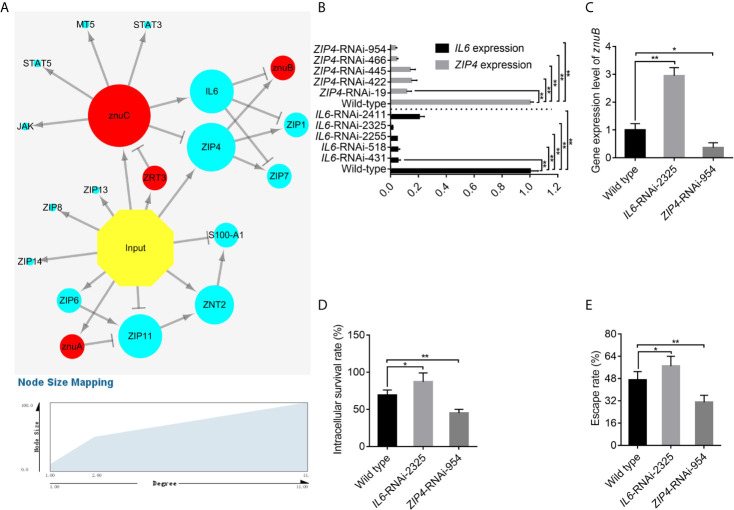
IL6 and ZIP4 play key roles in the trade-off for Zinc (Zn) between *Epinephelus coioides* and *Pseudomonas plecoglossicida*. **(A)** Predicted regulation network among *E. coioides* Zn nutritional immunity related genes and *P. plecoglossicida* Zn acquisition genes. Occurrence of infection, *E. coioides* genes and *P. plecoglossicida* genes are colored in yellow, blue and red, respectively. Positive and negative correlations are represented by lines. **(B)** To characterize the importance of *IL6* and *ZIP4*, five *IL6*-RNAi and *ZIP4*-RNAi *E. coioides* macrophages were constructed, and the silencing efficiencies were validated by quantitative real time PCR (qRT-PCR). **(C)** According to the pathogen-host gene regulatory network, *IL6* and *ZIP4* were supposed to promote and repress *znuB* expression, respectively. To validate this, qRT-PCR was carried out on wild type, *IL6*-RNAi-2325 and *ZIP4*-RNAi-954 *E. coioides* macrophages. **(D, E)** In order to validate whether the *IL6 and ZIP4* in *E. coioides* macrophages is important for intracellular killing of *P. plecoglossicida*, the intracellular viability and immune escape of wild type *P. plecoglossicida* was compared in wild type, *IL6*-RNAi-2325 and *ZIP4*-RNAi-954 *E. coioides* macrophages. Values are mean ± SD (n = 3, *P < 0.05, **P < 0.01).

Using dual RNA-seq, researchers could predict essential and also indirect interactions between pathogen and host. These analyses and predictions can provide clues for our research. Thus, 15 Zn nutritional immunity related genes identified in *E. coioides* (including *IL6*, *JAK*, *MT5*, *S100-A1*, *STAT3*, *STAT5*, *ZIP1*, *ZIP4*, *ZIP6*, *ZIP7*, *ZIP8*, *ZIP11*, *ZIP13*, *ZIP14*, and *ZNT2*) and 4 DEGs related to Zn acquisition found in *P. plecoglossicida* (including *ZRT3*, *znuA*, *znuB* and *znuC*) were used to predict a pathogen-host gene regulatory network (**Figure 5A**). From the perspective of correlations between bacterial Zn acquisition- and host Zn nutritional immunity-related genes, first, infection induced most Zn nutritional immunity- and bacterial Zn acquisition-related genes. Secondly, there were interactions between bacteria and host genes. Zn acquisition related genes such as *znuC* and *znuA* could influence host Zn nutritional immunity, thereby activating *IL6* expression or repressing *ZIP4* and *ZIP11* expression. Meanwhile, *IL6* and *ZIP4* could repress and induce the expression of *znuB*, respectively. Finally, there was a mutual regulatory relationship between host genes. For instance, *IL6* inhibited *ZIP1* and *ZIP7*; *ZIP4* activated *ZIP1* and *ZIP7*; *ZIP6* induced *ZIP11*, *ZNT2*, and *S100-A1*. In this interaction network, *IL6* and *ZIP4* were in the center of the battlefield, suggesting that they had critical roles in pathogen-host interactions.

### Effects of *IL6* and *ZIP4* on *P. plecoglossicida* Survival in Macrophages

According to previous study, *P. plecoglossicida* are phagocytosed by macrophages in the spleen of the infected fish, while it is proved to be capable of intracellular survival and replication (72, 73). Macrophages possess numerous mechanisms to combat microbial invasion, including sequestration of essential nutrients, like Zn (50). As we have shown in **Figure 1C**, increased Zn uptake in the Golgi apparatus of *E. coioides* macrophages after infection was observed, which indicated the existence of Zn nutritional immunity in macrophages induced by *P. plecoglossicida* infection. Therefore, we speculated that Zn nutritional immunity in macrophages may affect the intracellular survival of *P. plecoglossicida*, and further study was carried out on *E. coioides* macrophages to investigate the host-pathogen interaction during Zn nutritional immunity.

To characterize the importance of *IL6* and *ZIP4*, five *IL6*-RNAi and *ZIP4*-RNAi *E. coioides* macrophages were constructed, and the silencing efficiencies were validated by qRT-PCR. *IL6* and *ZIP4* levels in *E. coioides* macrophages treated with RNAi were significantly reduced (**Figure 5B**). Among them, *IL6*-RNAi-2325 and *ZIP4*-RNAi-954 with the best gene silencing efficiencies were selected for further research.

According to the pathogen-host gene regulatory network, *IL6* and *ZIP4* were supposed to promote and repress *znuB* expression, respectively. To validate this, qRT-PCR was carried out on wild type, *IL6*-RNAi-2325 and *ZIP4*-RNAi-954 *E. coioides* macrophages. Our results showed that, the silencing of *IL6* and *ZIP4* significantly increased and decreased *znuB* expression, respectively, which confirmed the above pathogen-host gene regulatory prediction network (**Figure 5C**).

Furthermore, in order to validate whether the *IL6 and ZIP4* in *E. coioides* macrophages is important for intracellular killing of *P. plecoglossicida*, the intracellular viability and immune escape of wild type *P. plecoglossicida* was compared in wild type, *IL6*-RNAi-2325 and *ZIP4*-RNAi-954 *E. coioides* macrophages. As depicted in **Figures 5D, E**, compared with WT macrophages, viability and immune escape of *P. plecoglossicida* in *IL6* and *ZIP4* silenced macrophages were markedly enhanced and decreased, respectively.

Taken together, these findings illuminated an important role for *IL6 and ZIP4* in intracellular killing of *P. plecoglossicida*. Some physiological property of the phagocytic cell that increases metal availability is changing after the silence of *IL6 and ZIP4*.

### Fur Negatively Regulates the Expression of *znuCBA* in *P. plecoglossicida*

**Figure 6:**
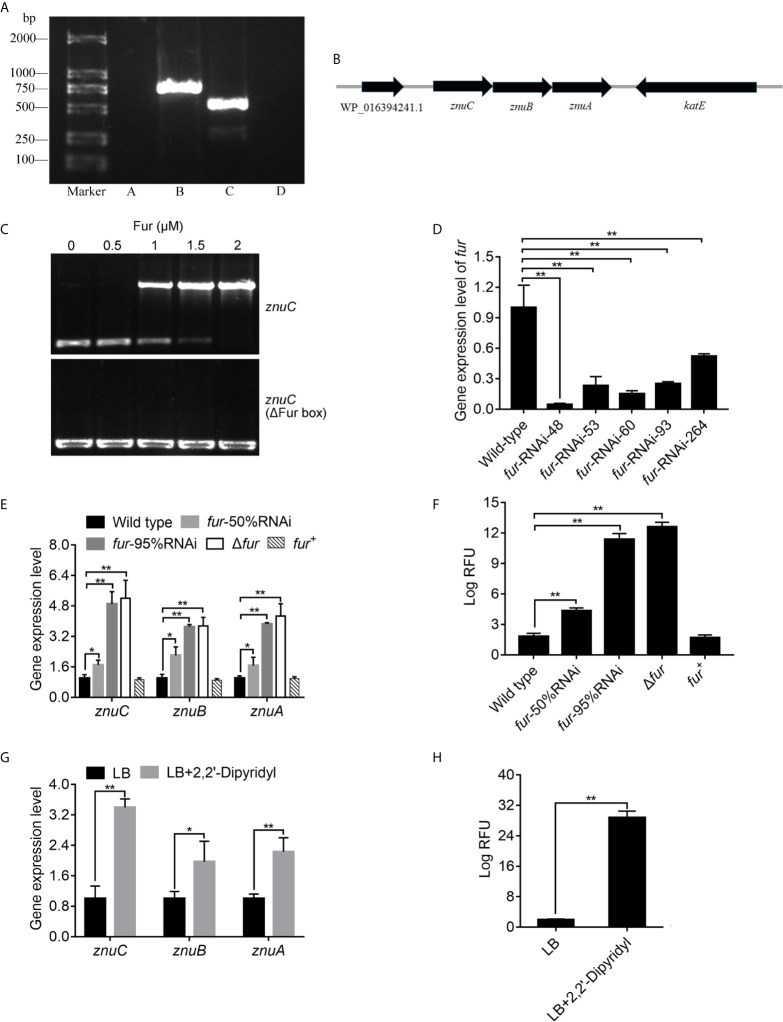
*ZnuCBA* is negatively regulated by Fur. **(A)** In order to validate whether *znuA*, *znuB* and *znuC* were transcribed into a single mRNA molecule, reverse transcription PCR (RT-PCR) was carried out to assess RNA isolated from *Pseudomonas plecoglossicida* using four pairs of primers. Pair A was designed to amplify the region between *WP_016394241.1* and *znuC*, pair B between *znuC* and *znuB*, pair C between *znuB* and *znuA*, and pair D between *znuA* and *katE*. Primer pairs A and D produced no bands, while primer pairs B and C produced bands of correct sizes. **(B)** Genetic organization of the *znuCBA* operon. **(C)** To validate the binding of Fur on the *znuC* promoter, the electrophoretic mobility shift assay (EMSA) was carried out. 6-carboxyfluorescein-labeled DNA fragment from the promoter region of *znuC* (upper panel) or DNA fragment with the same boundaries but with Fur box removal (lower panel) was added to the reaction mixture containing different concentrations of the Fur protein. **(D)** In order to assess the function of Fur, five *fur*-RNAi strains were constructed, and the silencing efficiencies were validated by quantitative real time PCR (qRT-PCR). **(E)** To validate whether the Fur in *P. plecoglossicida* is important for the regulation of *znuCBA*, qRT-PCR was carried out in wild type, *fur*-50%RNAi, *fur*-95%RNAi, Δ*fur* and *fur* ^+^ strains. **(F)** For further validating *znuCBA* downregulation via Fur, a *znuCBA*-reporter gene fusion controlled by the putative Fur promoter (nt -900 to +150) was constructed. Expression was assessed by measuring fluorescence with *fur* knocked down or knocked out. **(G)** To validate the effect of Iron (Fe) limitation on *znuCBA* expression, expression of *znuCBA* in wild type were compared in LB and Fe-limiting condition with 2’,2-Dipyridyl. **(H)** For further validating *znuCBA* upregulation under Fe limitation, transcription levels of the *znuC*-GFP reporter gene fusion in *P. plecoglossicida* under Fe limiting conditions was detected. Values are mean ± SD (n = 3, *P < 0.05, **P < 0.01).

The three genes, *znuA*, *znuB* and *znuC* in *E. coli* are transcribed into a single mRNA molecule (74). In order to validate this in *P. plecoglossicida*, we performed RT-PCR to assess RNA isolated from *P. plecoglossicida* using primers spanning adjacent genes, and *znuC, znuB* and *znuA*, flanked by *WP_016394241.1* and *katE*, were transcribed into one operon as *znuCBA* (**Figures 6A, B**). This corroborated previously reported studies, and indicated that these three genes may be controlled by the promoter upstream of *znuC*.

In order to investigate the regulation mechanism of *znuCBA* in *P. plecoglossicida*, Virtual Footprint was used to analysis the promoter of *znuC*. Virtual Footprint indicated a Fur putative binding site (5’-TGAATACT-3’) located within nt -545 to -538 upstream the start codon of *znuC*. To validate the binding of Fur on the *znuC* promoter, the EMSA assay was carried out. The EMSA assay (**Figure 6C**) revealed that Fur was able to strongly interact with a 364-bp DNA fragment (nt -361 to +3; part of the *znuC* promoter region), but not with another DNA fragment with the same boundaries after Fur binding site (nt -545 and -538) removal.

In order to assess the function of Fur, we constructed a *fur* null mutant Δ*fur*, as well as a complemented strain *fur* ^+^, and identified them by PCR (**Figure S4**). Meanwhile, five *fur*-RNAi strains were constructed, and the silencing efficiencies were validated by qRT-PCR (**Figure 6D**). Then, *fur*-RNAi strains with approximately 50% (*fur*-RNAi-264) and 95% (*fur*-RNAi-48) silencing efficiencies for *fur* were used for further research. To validate whether the Fur in *P. plecoglossicida* is important for the regulation of *znuCBA*, qRT-PCR was carried out in wild type, *fur*-50%RNAi, *fur*-95%RNAi, Δ*fur* and*fur* ^+^ strains. The Δ*fur* mutant displayed a significantly higher *znuCBA* expression than wild type (**Figure 6E**), while the high expression of *znuCBA* could be complemented by ectopic expression of *fur* (**Figure 6E**). After *fur* knockdown, *P. plecoglossicida* showed significantly increased *znuCBA* expression in a *fur*-dependent manner (**Figure 6E**).

For further validating *znuCBA* downregulation via Fur, a *znuCBA*-reporter gene fusion controlled by the putative Fur promoter (nt -900 to +150) was constructed. A significant increase of *znuCBA*-GFP activity was observed in *fur* knockout and knockdown strains, and complemented by ectopic expression of *fur* (**Figure 6F**). Simultaneously, the *znuCBA* expression (**Figure 6G**) and *znuCBA*-GFP activity (**Figure 6H**) of *P. plecoglossicida* significantly increased when cultured under Fe limiting conditions.

Taken together, these results suggested that Fur was a transcriptional regulator targeting and regulating the *znuCBA* promoter.

## Discussion

**Figure 7:**
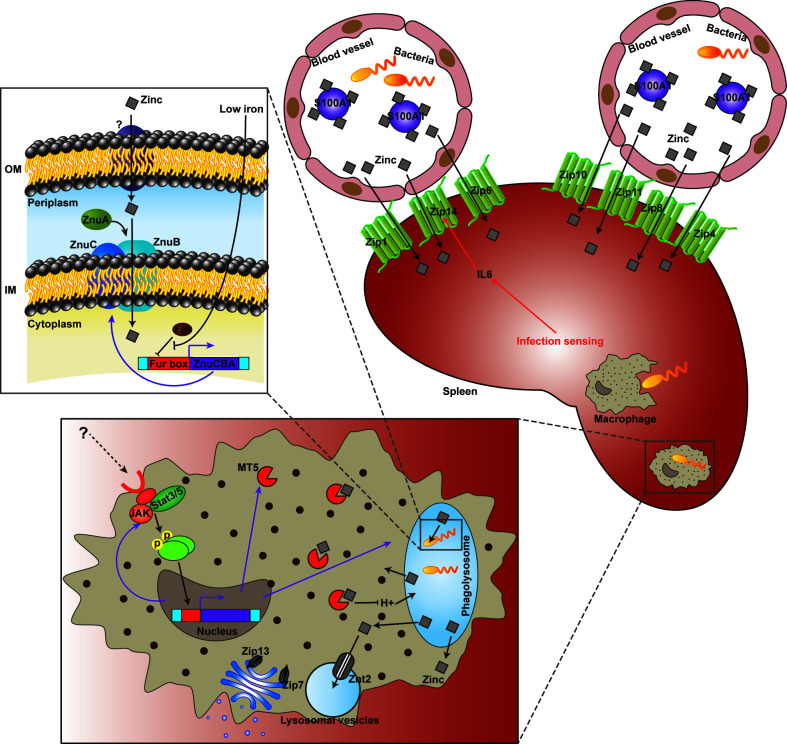
Working model of the struggle for Zinc (Zn) between *Epinephelus coioides* and *Pseudomonas plecoglossicida.*

Micronutrients including Fe, Zn and Mn are universally essential for life. In order to survive and proliferate, pathogenic bacteria must plunder micronutrients from infected host tissues. Taking advantage of pathogens’ requirement for micronutrients, mammals have developed a number of strategies to control bacterial infections via limiting microbial access to these micronutrients by evolving complex isolation mechanisms (75). However, research on fish nutritional immunity and its function in pathogen-host interactions remain scarce. What’s more, the understanding of fish Zn nutritional immunity is almost inexistent. The above results displayed Zn was withheld from serum and accumulated in the spleen after infection, which is probably a strategy that sequestrates Zn from the extracellular environment. Meanwhile, increased Zn uptake in the Golgi apparatus of macrophages after infection was also observed. Furthermore, 15 Zn nutritional immunity related genes were induced in *E. coioides* after *P. plecoglossicida* infection, including *IL6*, *JAK*, *MT5*, *S100-A1*, *STAT3*, *STAT5*, *ZIP1*, *ZIP4*, *ZIP6*, *ZIP7*, *ZIP8*, *ZIP11*, *ZIP13*, *ZIP14*, and *ZNT2*. *ZIP1*, *ZIP4*, *ZIP6*, *ZIP7*, *ZIP8*, *ZIP11*, *ZIP13*, *ZIP14*, and *ZNT2* belong to solute carrier 39A (SLC39A) Zn transporters (ZIPs) and solute carrier 30A (SLC30A) Zn transporters (ZnT), which are responsible for the homeostasis of Zn and other metals in mammalian species (76). Liuzzi et al. (77) demonstrated that the expression of *ZIP14* is positively regulated by IL-6, and this Zn transporter is likely to have a critical function in hypozincemia, which leads to acute phase reactions accompanied by infection and inflammation. The other genes are closely related to extracellular and intracellular Zn sequestration (4, 75), which is discussed in detail below. These results indicated the existence of *E. coioides* Zn nutritional immunity induced by *P. plecoglossicida* infection (**Figure 7**).

As a transition metal, Zn in living systems ranks second in content and is subject to strict control. Although Zn nutritional immunity remains unclear, in recent years, studies have begun to clarify the mechanism of Zn sequestration in organisms, organs, tissues and cells in mammals during bacterial infection (4, 5). The main mechanism of extracellular Zn restriction is through the heterodimer calprotectin composed of S100A8 and S100A9. Calprotectin limits the utilization of Zn by microorganisms in the local environment through binding of Zn with femtomola affinity (13). S100A7 can control the growth of microorganisms on the body surface, including mucous membranes, by isolating Zn (15). The roles of other members of the S100A family in infection control remain undefined, although they have been reported to perform Zn binding functions. In the present study, *S100-A1* was significantly induced in wild type strain-infected *E. coioides*, but downregulated in the *znuC-*95%RNAi strain group, which indicated that *S100-A1* is not only involved in Zn sequestration, but could also sense and response to the status of Zn acquisition in bacteria infecting fish (**Figure 7**).

Intracellular Zn nutritional immunity has been also reported in mammalian macrophages (78, 79). Phagolysosomes of macrophages containing pathogens may form a Zn-deficient environment. There is evidence that phagocytosed *C. albicans* yeasts upregulate ZRT2, a Zn transporter (80). Zn homeostasis in pathogen-containing macrophages is a very dynamic and strongly modulated process. Winters and colleagues (81) were the first to describe the opposite effects of GM-CSF and IL-4 on Zn homeostasis in macrophages with *H. capsulatum*. Subramanian Vignesh and collaborators (50) demonstrated that after inactivated macrophages undergo infection with *H. capsulatum*, Zn content in cells is high, indicating that without macrophage stimulation, *H. capsulatum* may obtain this key micronutrient any time. After treatment with GM-CSF, Zn was removed from phagocytosed yeasts and transported to the Golgi apparatus. This Zn migration in macrophages was accompanied by a decrease in Zn utilization by *H. capsulatum*. Subramanian Vignesh and co-workers (50) speculated that the regionalization of Zn in the Golgi apparatus might be due to the actions of ZnT4 and ZnT7, which are transcriptionally upregulated in *H. capsulatum* infected macrophages. These cationic efflux proteins transport Zn from the cytoplasm to subcellular organelles (or pump the metal out of cells), whereas Znt4 and ZnT7 were previously shown to be associated with Zn transport in other cell types (82, 83). In the present study, intra-Golgi compartmentalization of Zn was also observed, while *ZnT4* and *ZnT7* were not found in *E. coioides*. Instead, *Zip13*, another iron efflux protein encoding gene previously implicated in Golgi zinc transport (84), was upregulated in *P. plecoglossicida*-containing macrophages. Therefore, in *E. coioides*, Zip3 might be a zinc gatekeeper in the Golgi apparatus, which contributed to Zn sequestration from the cytosol into the Golgi apparatus (**Figure 7**).

In mammalian macrophages, the availability of Zn might be further constricted by Zn-binding metallothioneins (Mts), because Mt1 and Mt2 amounts STAT3/STAT5-dependently are increased, accompanied by Zn restriction. These phenomena were also related to increased production of ROS in the phagolysosome, which created a “perfect storm” of antibacterial effects. In the present study, *STAT3* and *STAT5* were both significantly induced, which might contribute to Zn restriction and ROS production in *E. coioides* macrophages. However, *Mt1* and *Mt2* were not found in *E. coioides*. Instead, *Mt5*, another Zn-binding metallothionein (85), was found to be upregulated as *STAT3* and *STAT5* in *P. plecoglossicida*-containing macrophages. Therefore, in *E. coioides*, MT5 might further limit the availability of Zn in a STAT3/STAT5-dependent manner. In addition, the transcription levels of ZNT2, a Zn transporter sequestering Zn into intracellular vesicles (such as lysosomal vesicles) for protection from Zn cytotoxicity, were increased. Hence, ZNT2 might be also involved in Zn homeostasis during the pathogen-host interaction (**Figure 7**), which deserves further investigation.

Although the host has Zn nutritional immunity, the pathogen can still reproduce in the infected host, which indicates that the pathogen has evolved an effective Zn scavenging strategy under the limitation of Zn nutritional immunity. For example, in *S. cerevisiae*, the Zn responsive transcription factor Zap1 addresses a decrease in metal amounts by upregulating the Zn importers Zrt1 and Zrt2 and the vacuolar Zn exporter Zrt3 (86–88). The Zn stored in vacuoles can be quickly transported to the cytosol through Zrt3 (89). Here, *ZRT3* was significantly increased in both wild type and *znuC*-RNAi *P. plecoglossicida* which infected *E. coioides*. This indicated that *ZRT3* might contribute to counteracting Zn nutritional immunity independent of *znuC*. Another important “neutralizer” against Zn nutritional immunity is the Zn high-affinity uptake system ZnuABC (28–30), which is responsible for importing Zn through the cytoplasmic membrane of bacteria (5, 27). ZnuABC has demonstrated roles in the pathogeneses of some bacterial infections. For example, ZnuABC has a critical function in *S. typhimurium* resistance to calprotectin-mediated Zn chelation in the intestine (20). *P. aeruginosa* relies on the znuABC Zn transporter to overcoming calprotectin-mediated growth inhibition (31). Interestingly, the *P. aeruginosa* intracellular zinc content is not affected by the dysfunction of the ZnuABC transporter evidently, which suggested a redundant mechanisms for the acquisition of Zn in *P. aeruginosa* at Zn limiting condition. Moreover, a study showed that ZnuABC is very important for bacteria to infect fish (66). Our previous study identified the ZnuCBA transporter in *P. plecoglossicida*, with all three components, including ZnuC (ATPase), ZnuB (inner membrane permease) and ZnuA (periplasmic zinc binding protein) (38). According to the current results, works done in other systems and homology, ZnuCBA was necessary for virulence in *P. plecoglossicida* by promoting Zn uptake. Furthermore, these 3 genes in *P. plecoglossicida* were transcribed into a single mRNA molecule and negatively regulated by a Fur binding box located upstream of *znuC* (**Figure 7**). Fur represents an important regulator of Fe absorption, and is closely related to oxidative stress. It can also regulate the expression of virulence genes by sensing changes in Fe concentration. During infection, vertebrates use the serum protein transferrin to chelate Fe, thereby forming a Fe-deficient environment. Many pathogenic bacteria perceive this Fe depletion as a signal in their vertebrate host and trigger the expression of pathogenic genes, so that they can adapt to the Fe-deficient environment (90). For example, Fur helps *Staphylococcus aureus* perceive changes in Fe concentration, thereby regulating the expression of many virulence factors, such as cytolysins and immunomodulatory proteins, to resist the attack of neutrophils (91). Fur also modulates the hemolysin system, which is widely distributed in pathogenic *Edwardsiella tarda*. The regulatory effect of Fur on ZnuCBA indicates a cross-talk between Zn and Fe acquisition against host nutritional immunity. This was not the first clue to such cross-talk. For example, in *Neisseria meningitidis*, the transport of Zn through the outer membrane is driven by the Zn-modulated TonB-dependent receptor ZnuD (24), which is similar to the *M. catarrhalis* heme transporter HumA in sequence. Z*nuD* expression in *E. coli* promotes the acquisition of heme (25). The dual regulation of ZnuD by Zur and Fur (Zn and Fe uptake regulators, respectively) further indicates ZnuD may be involved in the acquisition of Zn and heme (25). In addition, the cross regulation of ZnuD may be due to the increased demand for exogenous heme under Zn limitation, because some endogenous heme biosynthesis enzymes utilize Zn (26). Hence, the above results revealed a Fur and ZnuCBA dependent cross-talk between Zn and Fe acquisition against host nutritional immunity in *P. plecoglossicida*. The regulatory effect of Fur on ZnuCBA also indicated that sequestration of Fe in the host might occur before Zn sequestration. Microorganisms have a feature called “predictive adaptation”, which can predict events that will occur in their natural habitat (92). For example, *E. coli* exposed to lactose expresses genes related to the metabolisms of lactose and maltose simultaneously, predicting that when passing through the mammalian digestive tract, it will be sequentially exposed to both monosaccharides (93). For the same reason, when *P. plecoglossicida* Fur senses low Fe concentration, it predicts that after the sequestration of Fe, it will undergo Zn starvation, and optimally induces the expression of *znuCBA*. However, since ZnuD and Zur were not found in *P. plecoglossicida* genome, this cross-talk was independent of these molecules. In addition, which transporter is used by *P. plecoglossicida* to transport Zn through the outer membrane in the absence of ZnuD deserves further investigation.

Previous study by Pederick et al. (94) investigated the effect of Δ*znuA*-induced zinc limitation on the transcriptome of *P. aeruginosa*. Genes that enable adaptation to zinc limitation were significantly changed. Non-zinc-requiring paralogs of zinc-dependent proteins and a number of novel import pathways associated with zinc acquisition were significantly up-regulated. For example, *znuD* was dramatically increased by 172.2 folds in *P. aeruginosa* Δ*znuA*, which was proved to be involved in the acquisition of Zn and heme (25) but not found in *P. plecoglossicida* genome as we mentioned above. The Zn^2+^-independent transcription regulator DksA2 and P-type ATPase importer HmtA was transcriptionally increased by 134.6 and 5.6 folds in *P. aeruginosa* Δ*znuA* respectively (94), but not found in *P. plecoglossicida* genome. The cobaltochelatase subunit CobN and the biopolymer transport protein ExbD was transcriptionally increased by 122.2 and 44.7 folds in Δ*znuA* respectively (94), but not significantly affected in the present study. There are many other differences between the two studies like these. We also compared Mastropasqua et al. (95) with our study and found many similar differences. For example, *rpmE2*, *zmrABCD* and *xdhA* were significantly up-regulated in *P. aeruginosa* under zinc deficiency (95), while *rpmE2* and *zmrABCD* were not found in *P. plecoglossicida* genome, *xdhA* was down-regulated in *P. plecoglossicida*. These differences may be one of the reasons why the *P. aeruginosa* is more common in mammals infection and the *P. plecoglossicida* is more common in fish infection. On the other hand, these differentially expressed genes found by Pederick et al. (94) are predicted to be under the regulation of Zur, while the *P. plecoglossicida* do not have Zur, which may be an important reason for such a big difference in gene expression patterns under Zn restriction.

## Conclusion

In conclusion, the importance of ZnuC was revealed by investigating how *P. plecoglossicida* responds to Zn nutritional immunity during infection. While diverse Zn sequestration strategies against *P. plecoglossicida* have been observed in *E. coioides*, a common denominator of all these strategies is pushing *P. plecoglossicida* out of its ideal environment. With the importance of pathogen-host interaction continued to be reported, current and previous studies have revealed the importance of considering the host’s internal environment while evaluating the contribution of virulent genes.

## Data Availability Statement

The datasets presented in this study can be found in online repositories. The names of the repository/repositories and accession number(s) can be found in the article/**Supplementary Material**.

## Ethics Statement

The animal study was reviewed and approved by The Animal Ethics Committee of Jimei University (JMULAC201159).

## Author Contributions

All authors contributed to the article and approved the submitted version. QY and LH conceived the experiments. YZ, YQ, LZ and ML conducted the experiments. All authors assisted in the collection and interpretation of data. LH and QY wrote the manuscript.

## Funding

This work was supported by the Natural Science Foundation of Fujian Province (No. 2019J06020 and 2019J01695) and the “Distinguished Young Scientific Research Talents Plan in Universities of Fujian Province”.

## Conflict of Interest

The authors declare that the research was conducted in the absence of any commercial or financial relationships that could be construed as a potential conflict of interest.

## Supplementary Material

Supplementary Figure 1 Construction and confirmation of the knockout mutant strain Δ*znuC* and its complement *znuC* ^+^. WT, Amplification of wild-type using primers *znuC*_mut_-F-R; Δ*znuC*, Amplification of Δ*znuC* using primers *znuC*_mut_-F-R; *znuC* ^+^, Amplification of *znuC* ^+^ using primers *znuC*_mut_-F-R.

Supplementary Figure 2 KEGG pathway analysis of differentially expressed genes (DEGs) between wild-type *Pseudomonas plecoglossicida* and *znuC-*95%RNAi strain infected *Epinephelus coioides.* Spleen samples were assessed.

Supplementary Figure 3 KEGG pathway analysis of differentially expressed genes (DEGs) between wild-type and *znuC-*95%RNAi *Pseudomonas plecoglossicida* in the spleen of *Epinephelus coioides*.

Supplementary Figure 4 Construction and confirmation of the knockout mutant strain Δ*fur* and its complement *fur* ^+^. WT, Amplification of wild-type using primers *fur*_mut_-F-R; Δ*fur*, Amplification of Δ*fur* using primers *fur*_mut_-F-R, *fur* ^+^, Amplification of *fur* ^+^ using primers *fur*_mut_-F-R.

Supplementary Table 2 The summary of the number of mapped reads/input reads per replicate and the coverage.
