# Supplementary figures and images for: The Zinc Nutritional Immunity of Epinephelus coioides Contributes to the Importance of znuC During Pseudomonas plecoglossicida Infection
Source: Front Immunol. 2021 May 4;12:678699. doi: 10.3389/fimmu.2021.678699 (PMC8129501; doi:10.3389/fimmu.2021.678699)

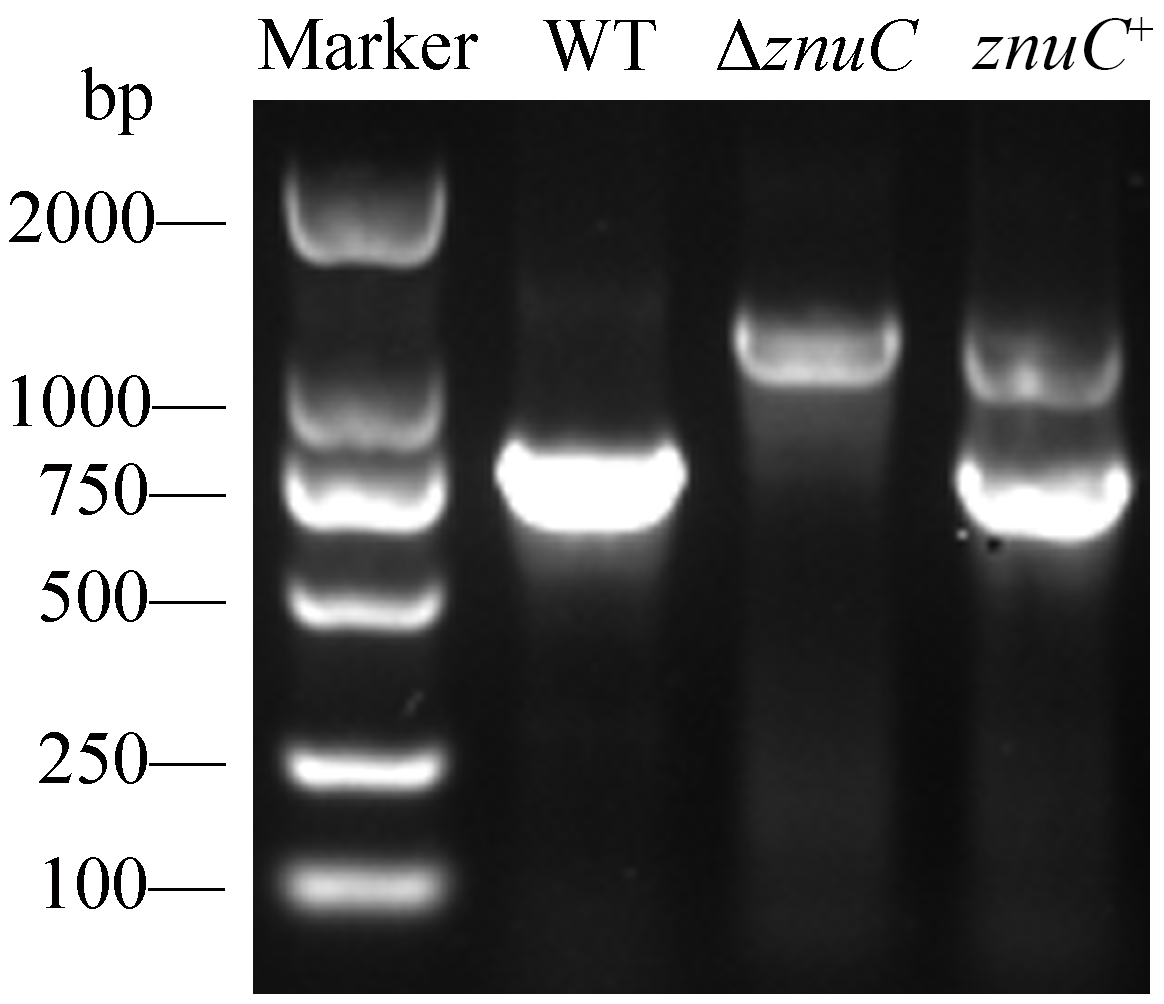

Supplement: Supplementary Figure 1 — Construction and confirmation of the knockout mutant strain ΔznuC and its complement znuC +. WT, Amplification of wild-type using primers znuC mut-F-R; ΔznuC, Amplification of ΔznuC using primers znuC mut-F-R; znuC +, Amplification of znuC + using primers znuC mut-F-R. [file Image_1.tif]

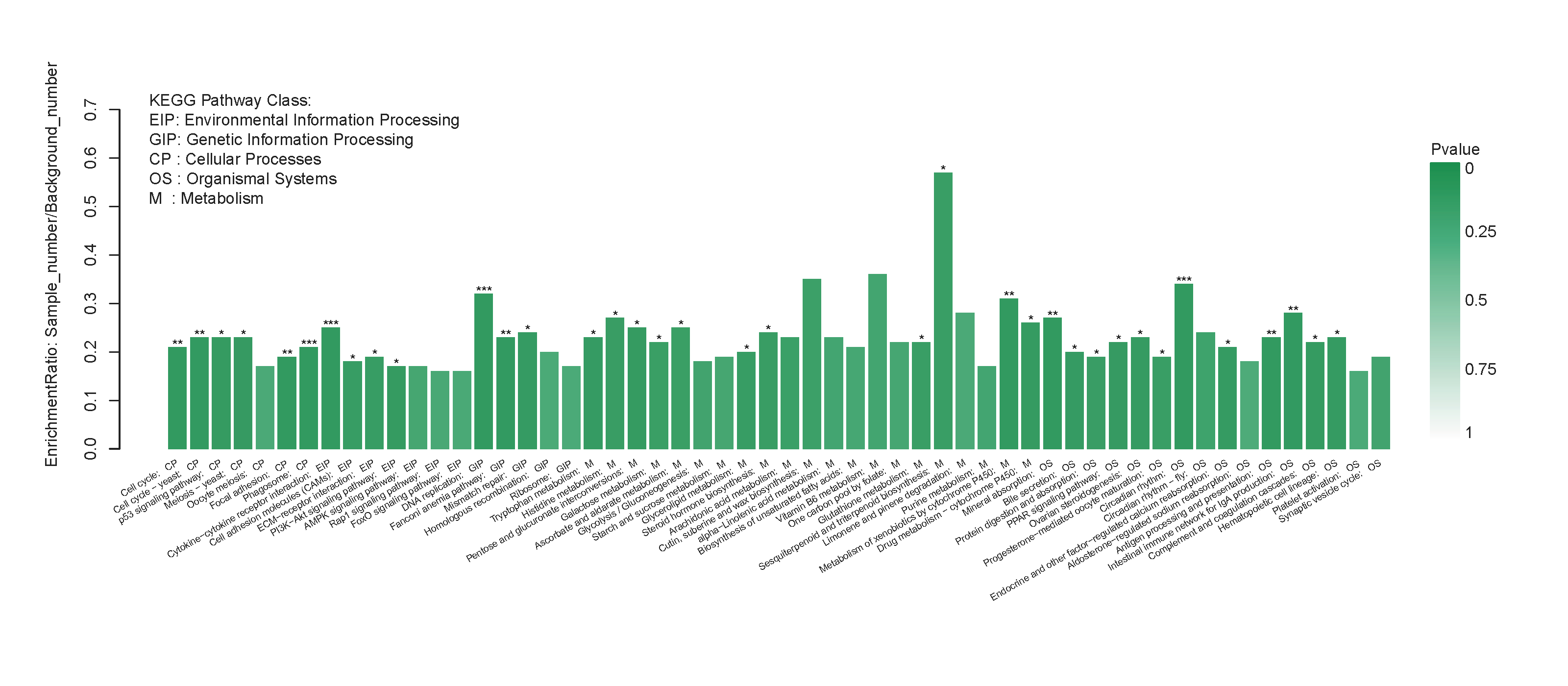

Supplement: Supplementary Figure 2 — KEGG pathway analysis of differentially expressed genes (DEGs) between wild-type Pseudomonas plecoglossicida and znuC-95%RNAi strain infected Epinephelus coioides. Spleen samples were assessed. [file Image_2.tiff]

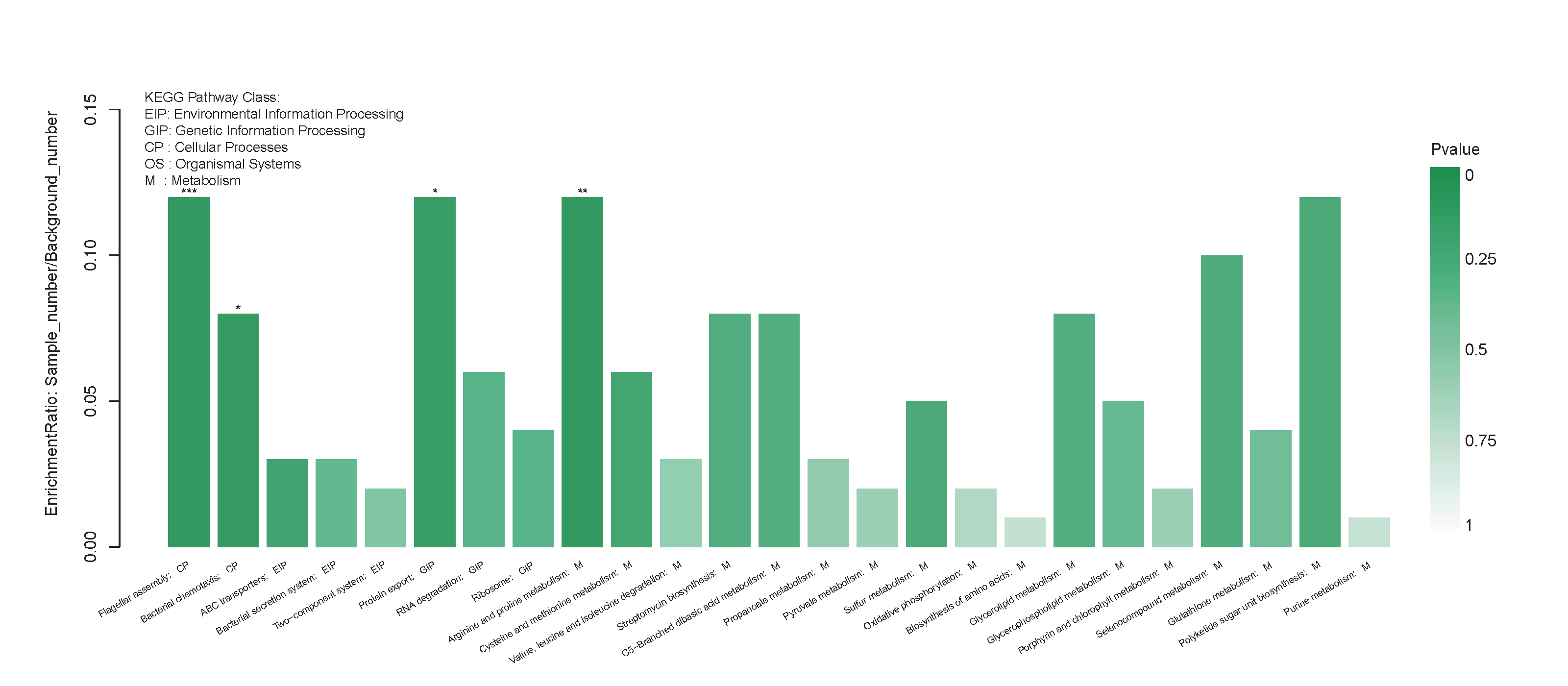

Supplement: Supplementary Figure 3 — KEGG pathway analysis of differentially expressed genes (DEGs) between wild-type and znuC-95%RNAi Pseudomonas plecoglossicida in the spleen of Epinephelus coioides. [file Image_3.tiff]

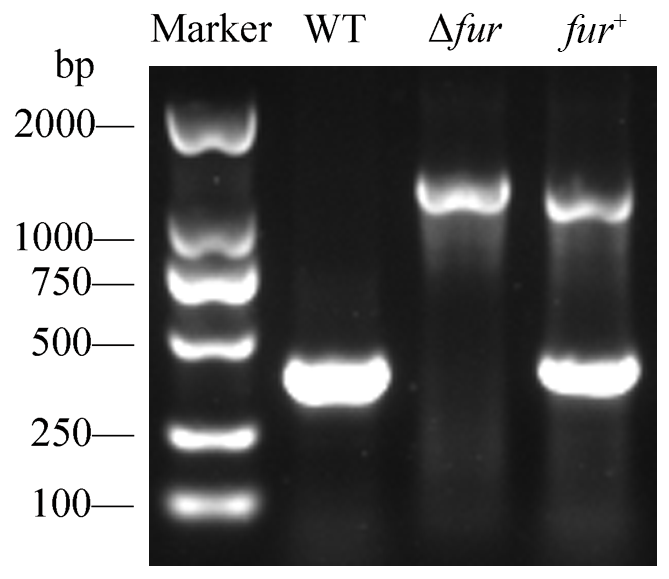

Supplement: Supplementary Figure 4 — Construction and confirmation of the knockout mutant strain Δfur and its complement fur +. WT, Amplification of wild-type using primers fur mut-F-R; Δfur, Amplification of Δfur using primers fur mut-F-R, fur +, Amplification of fur + using primers fur mut-F-R. [file Image_4.tif]
