# Supplementary material for: The Zinc Nutritional Immunity of Epinephelus coioides Contributes to the Importance of znuC During Pseudomonas plecoglossicida Infection
Source: Front Immunol. 2021 May 4;12:678699. doi: 10.3389/fimmu.2021.678699 (PMC8129501; doi:10.3389/fimmu.2021.678699)
Supplement: Supplementary file 5 [file Table_1.docx]

**Table S1. Primers used in the study**

|  | **Primers for the construction of the *znuC* GFP reporter gene system** |
| --- | --- |
| *znuC*-GFP-F | 5'-ATGAATTCCTTCTTCGTGGGAATGGCTGTCCTC-3' |
| *znuC*-GFP-R | 5'-ATAAGCTTGCGCGCACCAGGGTGGTCTTGCCGG-3' |
|  | **Primers for the construction of the mutants** |
| *znuC*_mut_-F | 5'-ATGAGTGACGCCCTGATCCGCCTCGGTGAAACCCAACATACCCCTGA-3' |
| *znuC*_mut_-R | 5'-TCAGCCATGCTTGCAGTGCTCTCCGTCAGCGATCGGCTCGTTG-3' |
| *fur*_mut_-F | 5'-ATGTCCATCACGCCGCTGGCCCACCGTGAAACCCAACATACCCCTGA-3' |
| *fur*_mut_-R | 5'-TCATGCTGCGCTCCGGCAGTTGCCGTCAGCGATCGGCTCGTTG-3' |
|  | **Primers for the qRT-PCR** |
| *znuC*-F | 5'-GCCCTGTCAGCGTTGCAGGAAGT-3' |
| *znuC*-R | 5'-CGCAGGCGGGTGATGAGGTTGT-3' |
| *znuB*-F | 5'-CTGCTCGGCATCCTTGCACCCA-3' |
| *znuB*-R | 5'-GCCTTCGACCATCGCCAGTTCCTC-3' |
| *znuA*-F | 5'-TCGCCCTGAAGACCACGGACA-3' |
| *znuA*-R | 5'-GCTGATGCGGAACCAATACCACC-3' |
| *ZRT3*-F | 5'-GCTGGTGATTGCGCTGGTGCTG-3' |
| *ZRT3*-R | 5'-AGCCGGTGATGCCCATTGCTG-3' |
| *fur*-F | 5'-GCACCGCATCGCCTCGCTCAAT-3' |
| *fur*-R | 5'-GTCGCTTATGCTGTCCTGTTCCAGTTCG-3' |
| *IL6*-F | 5'-GACCTCGACAATCCCAGCAC-3' |
| *IL6*-R | 5'-TAACGCCACCCAGTTTCCCT-3' |
| *JAK*-F | 5'-GACTGGGAGGTGTTCTGCGACTT-3' |
| *JAK*-R | 5'-TTCCACCGTGCAGTGACATG-3' |
| *MT5*-F | 5'-GGTAATACACTGGCAATGGTT-3' |
| *MT5*-R | 5'-GTGGTGGCTCCTATTGTTGA-3' |
| *S100-A1*-F | 5'-CACTGCTCACAGGCAATGGA-3' |
| *S100-A1*-R | 5'-AGTCACTCATCAAGGTGTTCCA-3' |
| *STAT3*-F | 5'-GTGACTCCTTATTGGCAGCAT-3' |
| *STAT3*-R | 5'-TGAAGCAAGACTTGAGTGGG-3' |
| *STAT5*-F | 5'-AAACCGCAATTTGTTACACTTC-3' |
| *STAT5*-R | 5'-GACTCACCAATTTAGTCGCATC-3' |
| *ZIP1*-F | 5'-AAACGCTGCTTTCTTCTGGC-3' |
| *ZIP1*-R | 5'-CCTTTCGCTCCTTCATGCTC-3' |
| *ZIP11*-F | 5'-CCCTGCTGGGGACTCTGTTC-3' |
| *ZIP11*-R | 5'-GGCGTGGTATCTTCACATCTAACTCTT-3' |
| *ZIP13*-F | 5'-GCTAGTGGCCGACGTGGTTT-3' |
| *ZIP13*-R | 5'-TGCCCGTTTCTGAGTGATGC-3' |
| *ZIP14*-F | 5'-GTAATGATCTGAGCCAGGGTA-3' |
| *ZIP14*-R | 5'-AATTTTGAGTCAGCTTGAGTGT-3' |
| *ZIP4*-F | 5'-GTGGTATGTCTGTCCGCAAGG-3' |
| *ZIP4*-R | 5'-ACCCGCTCAGAAGACCGATG-3' |
| *ZIP6*-F | 5'-AAGAAGAAGAACACCAGGAGAAA-3' |
| *ZIP6*-R | 5'-GGAGGGATCATCACAACACTTTA-3' |
| *ZIP7*-F | 5'-CATGATCACGGGCACGCACA-3' |
| *ZIP7*-R | 5'-GGATCAGAAACAGGATGAGGAAA-3' |
| *ZIP8*-F | 5'-TGTGATTAGTTCAAAGGAGCAT-3' |
| *ZIP8*-R | 5'-GCTAAATTGAGACTGTAATACGTGGATA-3' |
| *ZNT2*-F | 5'-GGCTTCCAGGAGAACAAATA-3' |
| *ZNT2*-R | 5'-CAATGATGATGATGATGGTGATGAT-3' |
| *gyrB*-F | 5'-CATCACCGTCATCATTCACCC-3' |
| *gyrB*-R | 5'-TTGACCACCGACACGCCTAC-3' |
| *gapdh*-F | 5'-GCCAAGGCTGTAGGCAAAGT-3' |
| *gapdh*-R | 5'-CGTCAAAGATGGATGAACGG-3' |
|  | **siRNA for gene silence** |
| *IL6*-431 | 5'-CCTGTAGTAACTCGGAAAT-3' |
| *IL6*-518 | 5'-CCCAGTTAACTGATCGCTT-3' |
| *IL6*-2255 | 5'-GAGGTCAACTAATCGCTTT-3' |
| *IL6*-2325 | 5'-GCAACAAGTTCACTCACTT-3' |
| *IL6*-2411 | 5'-GCAACATGTTCACTACTTT-3' |
| *ZIP4*-19 | 5'-GCCTCTACCTGAGAAGGTA-3' |
| *ZIP4*-422 | 5'-GCTACACCTGTCTGACAAT-3' |
| *ZIP4*-445 | 5'-GCACCTTATAGAGGCACAA-3' |
| *ZIP4*-466 | 5'-GCAACCTGACTGAATTAAT-3' |
| *ZIP4*-954 | 5'-GGCATTATCTGAATCAGTT-3' |
| *Fur*-48 | 5'-GCCTCGAGTCAAGATCCTT-3' |
| *Fur*-53 | 5'-GAGTCAAGATCCTTCAGAT-3' |
| *Fur*-60 | 5'-GATCCTTCAGATGCTCGAT-3' |
| *Fur*-93 | 5'-TCACATGAGTGCCGAGGAT-3' |
| *Fur*-264 | 5'-CCATATGGTCAACGTGGAG-3' |
|  | **shRNA for gene silence** |
| *znuC*-60-F | 5'-GAT CCT CTC GAC AGC ATC GAC TTG TTC AAG AGA CAA GTC GAT GCT GTC GAG ATT TTT TGC ATG-3' |
| *znuC*-60-R | 5'-CAA AAA ATC TCG ACA GCA TCG ACT TGT CTC TTG AAC AAG TCG ATG CTG TCG AGA G-3' |
| *znuC*-148-F | 5'-GAT CCG CAG TAC TGG GTT TGC TCA TTC AAG AGA TGA GCA AAC CCA GTA CTG CTT TTT TGC ATG-3' |
| *znuC*-148-R | 5'-CAA AAA AGC AGT ACT GGG TTT GCT CAT CTC TTG AAT GAG CAA ACC CAG TAC TGC G-3' |
| *znuC*-349-F | 5'-GAT CCA GCC CGA TCC AGA CCA TTT TTC AAG AGA AAA TGG TCT GGA TCG GGC TTT TTT TGC ATG-3' |
| *znuC*-349-R | 5'-CAA AAA AAG CCC GAT CCA GAC CAT TTT CTC TTG AAA AAT GGT CTG GAT CGG GCT G-3' |
| *znuC*-467-F | 5'-GAT CCG CCA GAC CGA GTT GTA CAA TTC AAG AGA TTG TAC AAC TCG GTC TGG CTT TTT TGC ATG-3' |
| *znuC*-467-R | 5'-CAA AAA AGC CAG ACC GAG TTG TAC AAT CTC TTG AAT TGT ACA ACT CGG TCT GGC G-3' |
| *znuC*-473-F | 5'-GAT CCC CGA GTT GTA CAA CCT CAT TTC AAG AGA ATG AGG TTG TAC AAC TCG GTT TTT TGC ATG-3' |
| *znuC*-473-R | 5'-CAA AAA ACC GAG TTG TAC AAC CTC ATT CTC TTG AAA TGA GGT TGT ACA ACT CGG G-3' |
