# Supplementary material for: The Zinc Nutritional Immunity of Epinephelus coioides Contributes to the Importance of znuC During Pseudomonas plecoglossicida Infection
Source: Front Immunol. 2021 May 4;12:678699. doi: 10.3389/fimmu.2021.678699 (PMC8129501; doi:10.3389/fimmu.2021.678699)

**Mapping results of *P. plecoglossida***

| **Sample** | **Mapped reads/Input reads** |
| --- | --- |
| WT-*in vitro*-1 | 18,849,268/18,956,364 |
| WT-*in vitro*-2 | 19,145,008/19,269,320 |
| WT-*in vitro*-3 | 18,755,216/18,877,038 |
| WT-*in vivo*-1 | 234,759/78,615,324 |
| WT-*in vivo*-2 | 43,993/91,029,522 |
| WT-*in vivo*-3 | 50,297/86,025,396 |
| *znuC*-95%RNAi-*in vivo*-1 | 6,568/101,644,502 |
| *znuC*-95%RNAi-*in vivo*-2 | 284,110/98,770,444 |
| *znuC*-95%RNAi-*in vivo*-3 | 2,710/102,629,284 |

**Mapping results of *E. coioides***

| **Sample** | **Mapped reads/Input reads** |
| --- | --- |
| Negative control-1 | 30,925,116/50,155,602 |
| Negative control-2 | 26,496,052/42,710,654 |
| Negative control-3 | 29,358,472/46,863,568 |
| WT-infection-1 | 54,982,522/78,615,324 |
| WT-infection-2 | 61,924,110/91,029,522 |
| WT-infection-3 | 59,840,286/86,025,396 |
| *znuC*-95%RNAi-infection-1 | 68,640,680/101,644,502 |
| *znuC*-95%RNAi-infection-2 | 67,842,636/98,770,444 |
| *znuC*-95%RNAi-infection-3 | 70,047,442/102,629,284 |

**Gene Body Coverage curves**


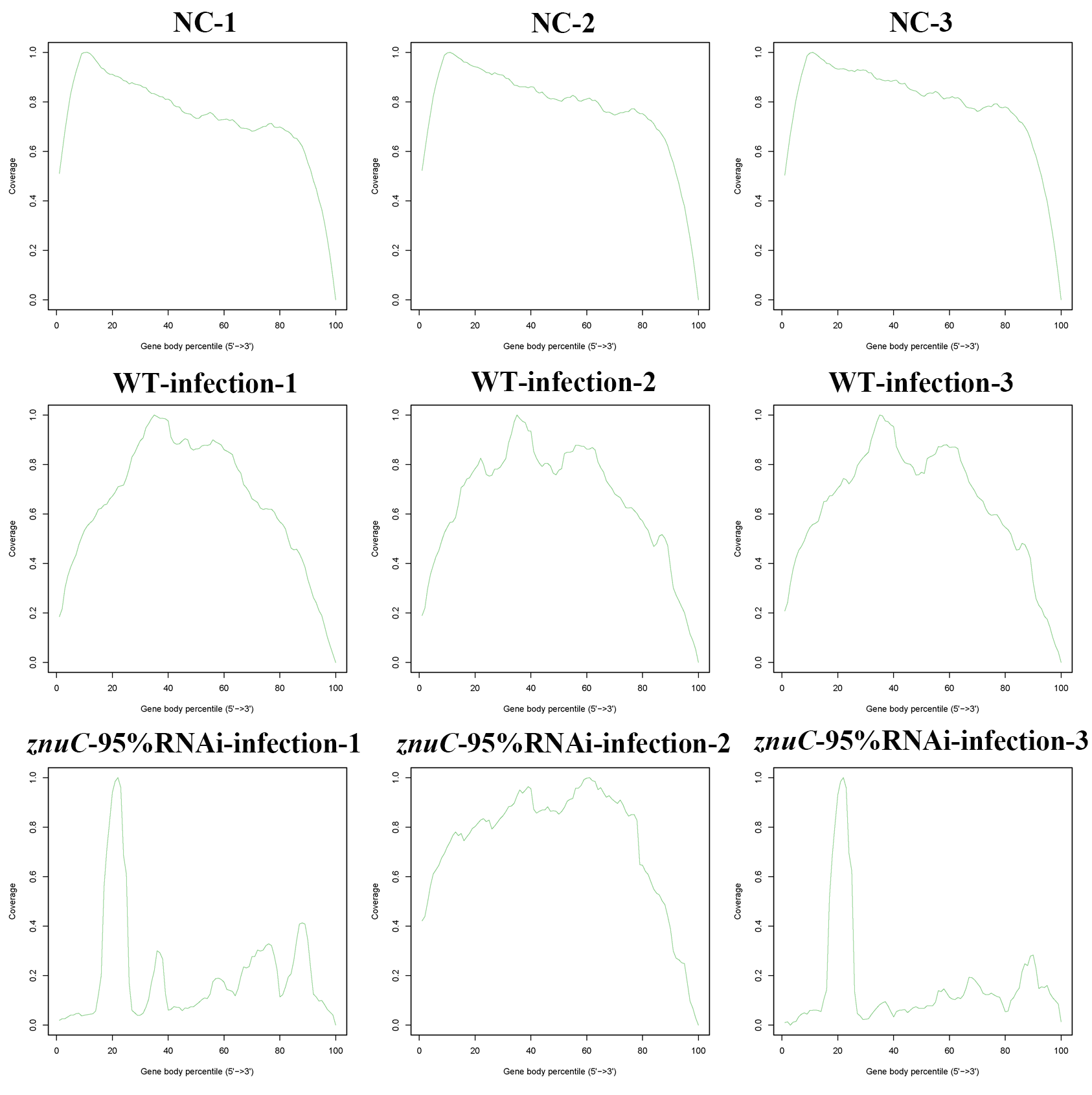

Supplement: Supplementary Table 2 — The summary of the number of mapped reads/input reads per replicate and the coverage. [file Table_2.docx]
